# Deep learning based automated diagnosis of bone metastases with SPECT thoracic bone images

**DOI:** 10.1038/s41598-021-83083-6

**Published:** 2021-02-19

**Authors:** Qiang Lin, Tongtong Li, Chuangui Cao, Yongchun Cao, Zhengxing Man, Haijun Wang

**Affiliations:** 1 School of Mathematics and Computer Science, Northwest Minzu University, No. 1, Xibei Xincun Rd., Lanzhou, 730030 Gansu China; 2 Key Laboratory of Streaming Data Computing and Applications, Northwest Minzu University, Lanzhou, 730030 Gansu China; 3 Key Laboratory of China’s Ethnic Languages and Information Technology of Ministry of Education, Northwest Minzu University, Lanzhou, 730030 Gansu China; 4 Department of Nuclear Medicine, Gansu Provincial Hospital, No. 204, Donggang Xilu Rd., Lanzhou, 730030 Gansu China

**Keywords:** Whole body imaging, Experimental models of disease

## Abstract

SPECT nuclear medicine imaging is widely used for treating, diagnosing, evaluating and preventing various serious diseases. The automated classification of medical images is becoming increasingly important in developing computer-aided diagnosis systems. Deep learning, particularly for the convolutional neural networks, has been widely applied to the classification of medical images. In order to reliably classify SPECT bone images for the automated diagnosis of metastasis on which the SPECT imaging solely focuses, in this paper, we present several deep classifiers based on the deep networks. Specifically, original SPECT images are cropped to extract the thoracic region, followed by a geometric transformation that contributes to augment the original data. We then construct deep classifiers based on the widely used deep networks including VGG, ResNet and DenseNet by fine-tuning their parameters and structures or self-defining new network structures. Experiments on a set of real-world SPECT bone images show that the proposed classifiers perform well in identifying bone metastasis with SPECT imaging. It achieves 0.9807, 0.9900, 0.9830, 0.9890, 0.9802 and 0.9933 for accuracy, precision, recall, specificity, F-1 score and AUC, respectively, on the test samples from the augmented dataset without normalization.

## Introduction

In the medical imaging field, *nuclear medicine imaging* is widely used for diagnosing, treating, evaluating, and preventing different diseases and medical conditions. It is different from the conventional structural imaging modalities such as Computed Tomography (CT), Magnetic Resonance Imaging (MRI), and Ultrasound. Structural imaging modalities provide only anatomic information about an organ or body part. In particular, nuclear medicine imaging is capable of revealing functional and structural variations in organs and tissues of a body. As part of modern medicine, nuclear medicine imaging is dominant in oncology, neurology, and cardiology.

As one of the widely used techniques, *Single Photon Emission Computed Tomography* (SPECT) can provide insights into physiological processes of the areas of concerns by detecting trace concentrations of radioactively-labelled compounds, like *Positron Emission Tomography* (PET). For SPECT examination, imaging equipment captures the emitted gamma rays from radionuclides that were injected into a patient’s body in advance for visualizing the inside of the body in a non-invasive manner. There are three kinds of the common radiotracers for SPECT imaging: [99mTc] Sestamibi for myocardial perfusion, [99mTc] MDP (methylene diphosphonate) for bone scanning, and [99mTc] HMPAO (exametazime) for blood flow in a brain. It is reported that more than 18 million SPECT scans are conducted each year in the United States^1^.

Bones are clinically identified as the common sites of metastasis in various malignant tumors such as prostate and breast cancer. These occupying lesions are viewed as areas of the increased radioactivity called *hot spots* in SPECT bone imaging. Quantitative SPECT bone scanning has the potential for providing an accurate assessment of the stage and severity of a disease. As such, the automatic classification of images plays a crucial role in constructing computer-aided diagnosis (CAD) systems. In the domain of medical image analysis, classifying images refers to producing classification outputs for given input images that identify whether a disease presents or absents^2^. During the past decades, medical image classification has been one of the application areas in the traditional machine learning^3,4^ and deep learning^5–12^.

Classification of SPECT images is also a hot topic in the field of deep learning-based medical image analysis. The main objective of existing work is to automatically diagnose various diseases ranging from Alzheimer’s disease^13^, Parkinson’s disease^14–16^ and thyroid disease^17,18^ to cardiac disease^19^. The openly available Parkinson’s Progression Markers Initiative (PPMI) dataset (http://www.ppmi-info.org/) was frequently used in existing work^14–16^.

Classifying SPECT bone images for the automated diagnosis of bone metastasis, however, has not been examined yet. The possible reasons for this are triple-fold:

- A nuclear medicine imaging is always limited by its poor spatial resolution and a low signal-to-noise ratio, particularly for a whole-body SPECT bone scan. As such, it is challenging to identify the precise location of a lesion and its adjacent structures, though an abnormal area of the increased uptake is noted.
- A whole-body SPECT bone image often has more than one lesion with the same or different primary diseases, which result in quite difficulties for correctly diagnosing and properly estimating various diseases.
- The big datasets of SPECT bone scans are rarely available as a result of the rarity of diseases and patient privacy. In addition, imbalanced samples commonly occur in the dataset of the SPECT bone imaging because the distribution of the images depends heavily on the available patients with the type of diseases.

In this work, we intend to automatically diagnose metastasis in thoracic SPECT bone images where bone metastasis of various primary cancers frequently develop, by constructing deep learning based classifiers conducted on real-world data of SPECT bone scans. In particular, each original whole-body SPECT bone image is cropped to extract the thoracic area, followed by data preprocessing and data augmentation operations. Then we introduce a group of famous convolutional neural networks (CNNs) to develop deep classifiers that can automatically answer whether a bone metastasis presents in the given thoracic SPECT bone image or not. Last, we use the real-world SPECT bone images acquired from clinical examinations to validate the effectiveness and performance of the developed classifiers. Experimental results demonstrate that our classifiers work well on identifying metastasis in thoracic SPECT bone images, achieving a score of 0.9807, 0.9900, 0.9830, 0.9890, 0.9802 and 0.9933 for *accuracy*, *precision*, *recall*, *specificity*, *F*-1 *Score*, and AUC, respectively, on the test samples with augmentation.

The main contributions of this work are: First, we identify the research problem of SPECT imaging-based automated diagnosis of bone metastasis. To the best of our knowledge, this is the first work on deep learning-based medical image analysis. Second, we cast the problem of automated metastasis diagnosis as a classification of thoracic SPECT bone images and develop CNNs-based classifiers by using the capacity of automatically learning the feature representations from images. Last, we evaluate the developed deep classifiers by using a set of real-world SPECT bone images. Experimental results demonstrate that our classifiers perform well in identifying the metastasized SPECT bone images.

The rest of this paper is organized as follows. “Materials and methods” presents the data of SPECT bone scans and the proposed deep classifiers, followed by reporting our experiments on real-world data in “Results”. The last section concludes this work and points out future research directions.

## Materials and methods

### Dataset

With a Siemens SPECT ECAM imaging equipment, the SPECT bone images used in this work were collected as a result of diagnosing bone metastasis in the Department of Nuclear Medicine, Gansu Provincial Hospital in 2018. The distribution of the intravenous administration of a radiotracer (i.e., 925/740 MBq Tc-99m) to a patient was generated by the equipment in SPECT examination.

Patients from different departments may have different SPECT bone images, including surgery, radiology, respiratory, thoracic rheumatology, orthopedics, breast, and oncology. Inpatients are the majority of the patients without excluding a few of the outpatients. 251 patients aged 43–92 years in total were finally diagnosed with normal (n = 166, ≈ 66%) and metastasis (n = 85, ≈ 34%). The follow-up patients are not included in our dataset because the SPECT imaging is mainly conducted on the diagnosis of severe diseases.

**Figure 1:**
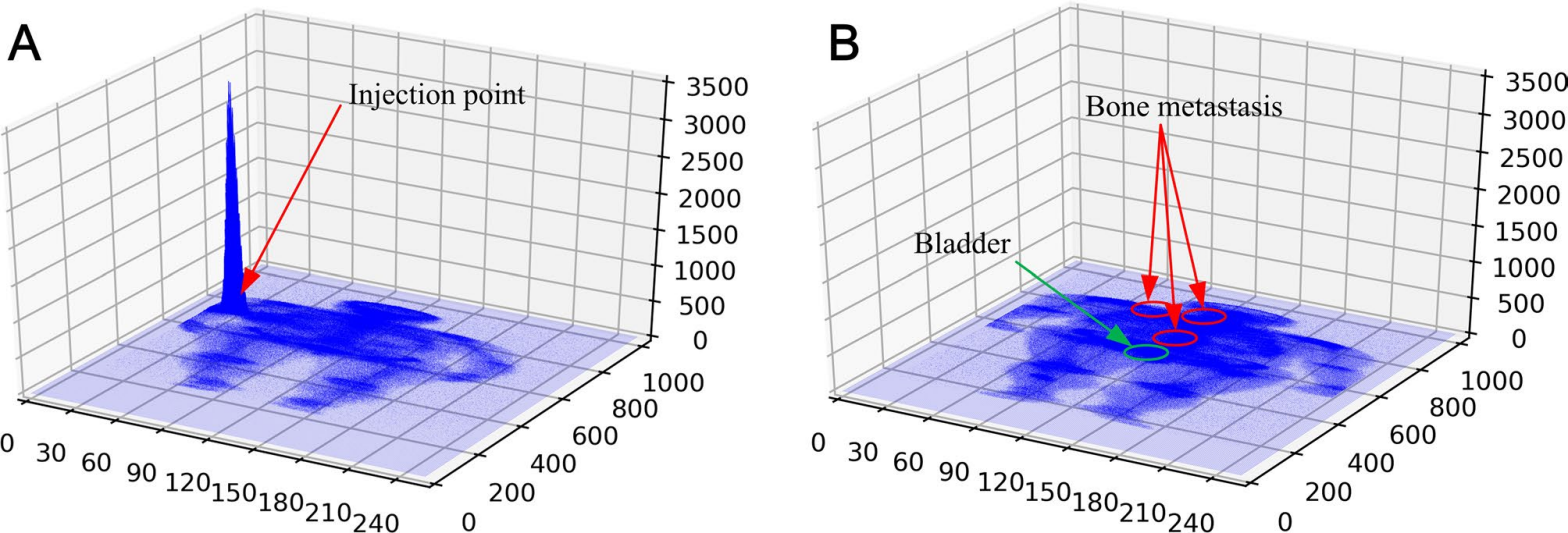
Distribution of radiation dosage in whole-body bone SPECT images. (**a**) Normal image with the concentrated injection point; and (**b**) image diagnosed with metastases marked by red polygons.

Generally, each SPECT examination records two images about anterior and posterior views if the examination is not damaged and or lost. Each SPECT bone image is stored in a DICOM file (.dcm). The image is a matrix of numbers to measure radiation dosage by using a 16-bit unsigned integer. Note that SPECT images that capture the various radiation within a wide dosage range are different from natural images with their pixel values ranging from 0 to 255. An image of a whole-body SPECT bone scan is 256 (width) × 1024 (height), visualizing most of the body of a patient. Figure 1 illustrates two whole-body SPECT bone images, where *x*-, *y*-, and *z*-axis denotes the width, height, and radiation dosage, respectively. The areas of increased uptake like injection point and bladder often confuse the true hot spots of bone metastasis with them if the traditional machine learning techniques are used for classifying the low-resolution SPECT bone images.

Formally, we use a matrix *BSI* to represent an image of whole-body SPECT bone image:

1 $$ BSI = \left[ {\begin{array}{*{20}l} {rd_{11} } & {rd_{12} } & \ldots & {rd_{1m} } \\ {rd_{21} } & {rd_{22} } & \cdots & {rd_{2m} } \\ \vdots & \vdots & \ddots & \vdots \\ {rd_{n1} } & {rd_{n2} } & \cdots & {rd_{nm} } \\ \end{array} } \right] $$

where *rd*_*ij*_ (1 ≤ *i* ≤ *m*, 1 ≤ *j* ≤ *n*) is the radiation dosage, with *m* = 256, and *n* = 1024 for the size of an image.

There are in total 346 sample images, i.e., the anterior and posterior, of whole-body SPECT bone scans from 251 patients in our dataset. In particular, our dataset consists of 220 normal samples and 126 samples diagnosed with metastasis. Table 1 provides the statistics of the original whole-body SPECT bone images, where the metastasized images are divided into three sub-categories according to the lesion location.

**Table 1:** Statistics of the image dataset of whole-body SPECT bone scan (n = 346).

	Normal	Metastasis		
		Multiple bone metastasis	Spinal metastasis	Metastasis outside spinal
Samples	220	111	14	1
Ratio	63.6%	32.1%	4.0%	0.3%

### Ethical approval

The study was approved by the Ethics Committee of Gansu Provincial Hospital (Lot No.: 2020-199). A requirement for the informed consent was waived for this study by the approval of Ethics Committee of Gansu Provincial Hospital. The fully anonymised image data was received by the authors on 28 August, 2020. The used bone SPECT images were de-identified before the authors received the data. We claim that all methods were carried out in accordance with relevant guidelines and regulations.

## Methodology

### Data preprocessing and augmentation

Since spine and ribs are the most common sites of metastases in a variety of cancers, the thoracic region will be cropped from each whole-body SPECT bone image to form the dataset of thoracic SPECT bone images in this work.

#### Cropping thoracic region

**Figure 2:**
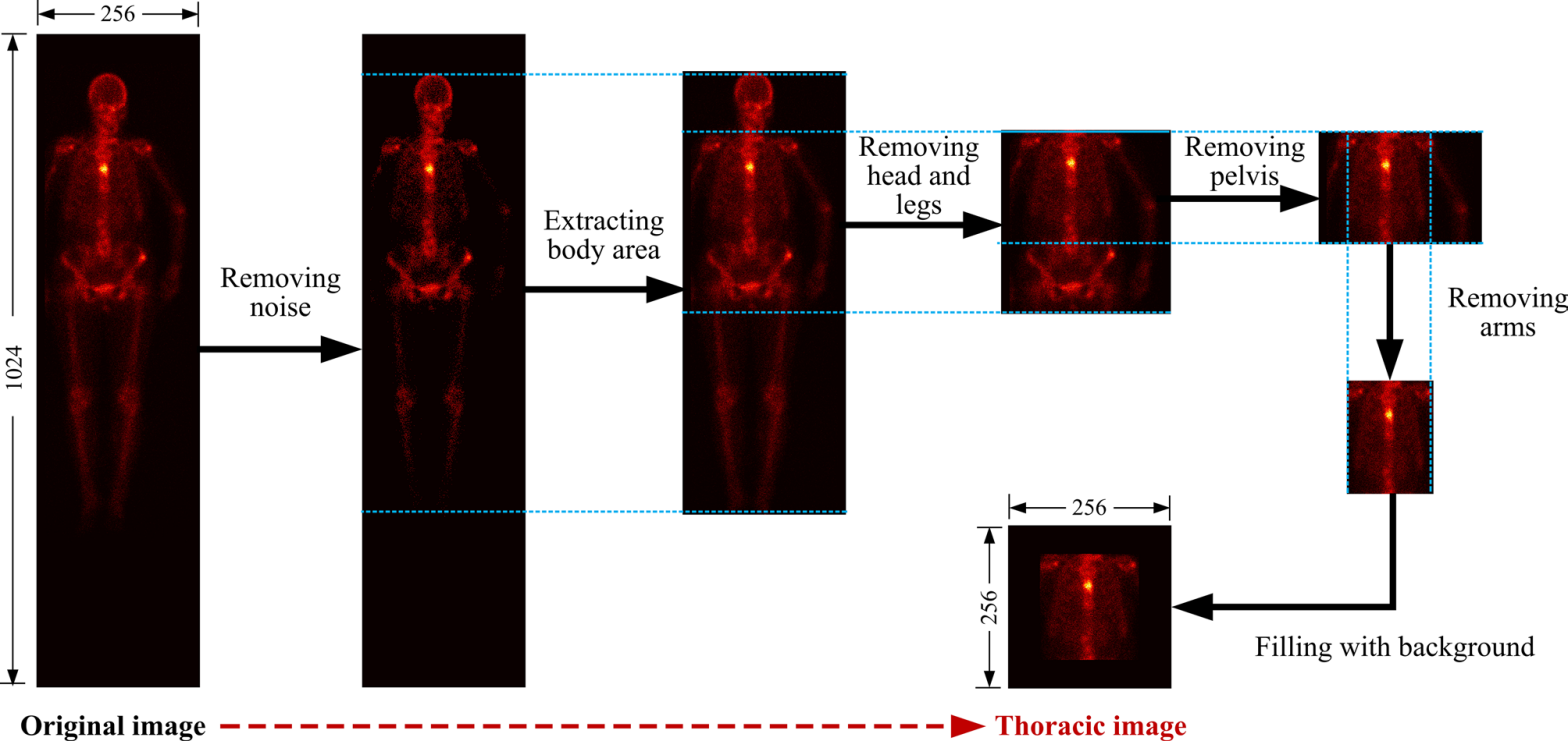
The process of cropping a 256 × 256 thoracic region from the original 256 × 1024 whole-body SPECT bone image.

Cropping thoracic region is for separating the spine and ribs from others in a 256 × 1024 image of whole-body SPECT bone scan, to obtain the thoracic imaging data. It is often challenging, however, to accurately separate parts of a body in a low-resolution SPECT bone image full of noise as a result of the wide ranges of radiation dosage. Traditional separation using the structure information of the skeleton performs mediocrely on separating whole-body SPECT bone images. On the contrary, the distribution of radiation dosage should be considered for adaptively cropping the thoracic region from an image of whole-body SPECT bone. Figure 2 depicts the process of cropping the thoracic region from an original image of 256 × 1024 whole-body SPECT bone, consisting of five stages as follows.

*Stage 1: Removing noise*. For a given image of whole-body SPECT bone with its size of 256 × 1024, we determine the maximum value of radiation dosage outside the body area by sweeping the image globally. For each image, such a maximum value is unique, which is thereby viewed as the threshold of noise of *thr*_*N*_. The elements in *BSI* with radiation dosage that are less than *thr*_*N*_ are set to 0, i.e., image background. The adaptive thresholding for different images can remove their noises while keeping the information of lesions.

*Stage 2: Extracting the body area.* After removing noise, the areas that are above the top of the head and below the toes in each image are further discarded. As a result, the remaining parts are the area of concerns.

**Figure 3:**
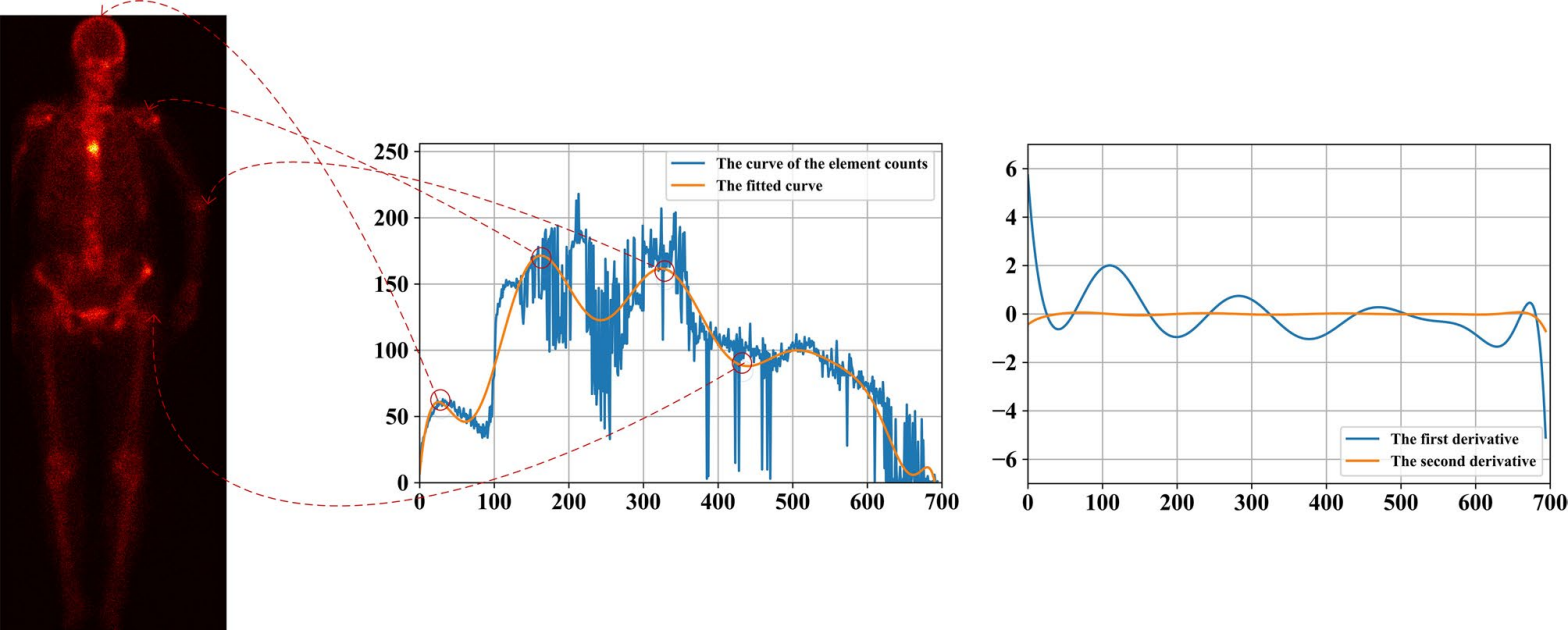
Cropping thoracic region based on curve fitting approach (left: the valid body area of a posterior SPECT image; middle: the fitted curve; and right: the curves of the first and second derivatives of the fitted curve.

*Stage 3: Removing head and legs.* For the extracted validated body area, we count the elements from the top down. The generated curve indicates the presence and intensity of radiation dosage so as to reveal parts of the body. As an example, the first three peak points of the fitted curve shown in Fig. 3 shows the beginning positions of the head, the right elbow, and the right shoulder. The third valley point shows the beginning position of the right leg.

*Stage 4: Removing pelvis.* The first and second derivatives plotted in Fig. 3 indicate the areas of arms, thorax, and pelvis. The thoracic region can then be extracted by relying on the facts that the width of the trunk is the almost same as the one of the area of legs, as well as the ratio of the height of a spine and that of a pelvis is about 3: 2.

*Stage 5: Filling with the background.* In order to reduce fitting errors, a randomly-determined penalty factor *σ* ∈ (5, 15) was introduced in the cropping process. The cropped thoracic region is enlarged to 256 × 256 by filling the rest of areas with its background. We call the cropped area as *thoracic SPECT image* or *thoracic image* in the following sections.

#### Thoracic image augmentation

In general, deep learning needs a big dataset. We thus apply a series of preprocessing operations on thoracic images to augment our dataset by considering the followings:

- It is inevitable that during a long-time SPECT scanning, the position and orientation of a patient are changed. A patient is often startled when the bed shifts to the next scanning position, for example. As a result, scanning may take up to 3 h. Therefore, classification models should be robust in dealing with the displacement and tilt in SPECT bone images.
- It is common to unsuccessfully record images in our dataset. An examination may have only the anterior view image and vice versa. This explains why there are only 346 images for 251 patients as reported in Table 1. Technical approaches are needed to handle the missing of SPECT bone images.

Data preprocessing techniques such as geometric transformations (i.e., image mirroring, translation, and rotation) and normalization are used for coping with the above problems. At the same time, the data in the dataset are extended.

#### Image mirror

**Figure 4:**
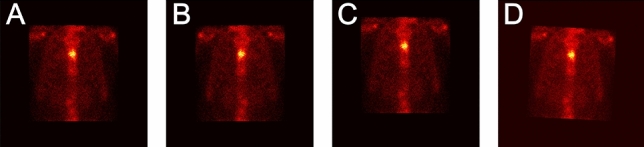
Examples of preprocessing a thoracic image with a hot pot of bone metastasis in the spine. (**a**) The original thoracic image; (**b**) the mirrored image; (**c**) the vertically translated image; and (**d**) the rotated image.

The horizontal mirror reverses a thoracic image right-to-left along the vertical center line of the image. For a given thoracic image as depicted in Fig. 4a, a mirrored counterpart of this image is shown in Fig. 4b.

#### Image translation

A thoracic image is translated by + *t* or − *t* pixels in either the horizontal or the vertical direction. For each thoracic image, the value of *t* is randomly assigned with an integer from the range [0, *t*_*T*_]. The value of *t*_*T*_ is statistically chosen based on the distribution of radiation dosage in all images. A value of 10 (4) for *t*_*T*_ is acceptable in the experiments for horizontal (vertical) translation. From Fig. 4c, we can see that the information about the hot spot in the translated image keeps perfectly.

#### Image rotation

A thoracic image is rotated by *r* degrees in either the left or right direction around its geometric center. For each thoracic image, *r* is randomly assigned with an integer within the range [0, *r*_*T*_]. Similarly, *r*_*T*_ is statistically determined by using the distribution of radiation dosage in all images and a value of 5° for *r*_*T*_ is set in our experiments. As an instance, Fig. 4d shows the rotated counterpart by randomly rotating the given image in Fig. 4a to the right direction by 3°.

#### Normalization (optional)

The min–max normalization technique is used to limit the wide range of radiation dosage of thoracic images to an interval [0, 1].

The generated images via preprocessing operations above are added to the original dataset in Table 1 to form our augmented dataset, which is outlined in Table 2. It is worth noticing only part of rather than the whole normal images were augmented in this work.

**Table 2:** Overview of the augmented data of thoracic images. (n = 2390).

	Normal	Metastasis		
		Multiple bone metastasis	Spinal metastasis	Metastasis outside spinal
Samples	1200	1043	133	14
Ratio	50.2%	43.6%	5.6%	0.6%

We organize the used data into three datasets *D*_1_–*D*_3_, with *D*_1_ denoting the original dataset, *D*_2_ and *D*_3_ denoting the augmented dataset with and without normalization respectively.

The subsequent section will describe the process of labelling thoracic SPECT bone images for obtaining ground truth in the experiments.

#### SPECT bone image annotation

The labelling image plays a key role in training reliable supervised classifiers. It is time-consuming, however, to label a SPECT image with the low spatial resolution. Relying on the online available tool called LabelMe^20^ by MIT, we developed an annotation system for labelling thoracic images in this work.

**Figure 5:**
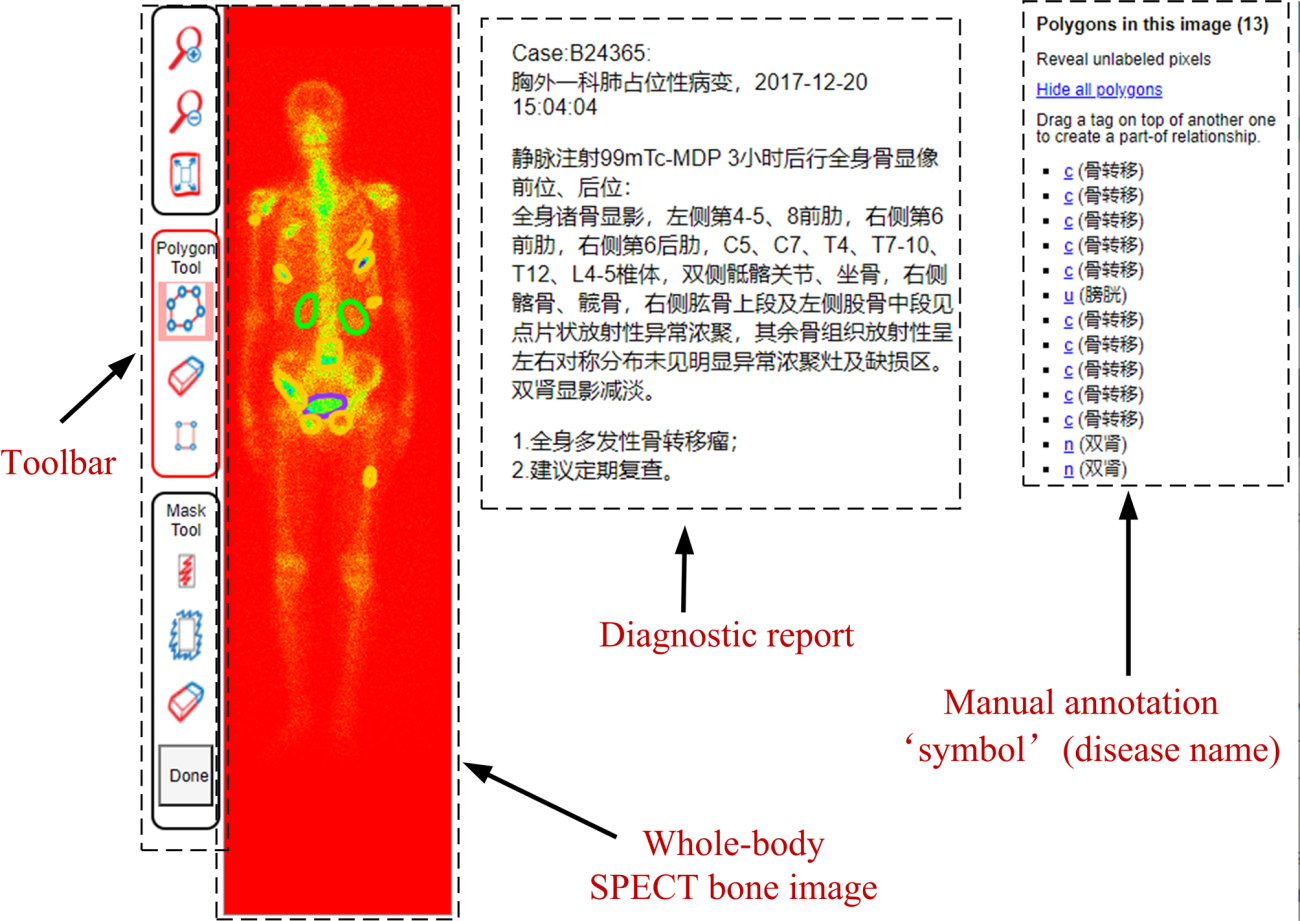
Labelling whole-body SPECT bone image using LabelMe (version 3.0, http://labelme2.csail.mit.edu/Release3.0/browserTools/php/sourcecode.php) based annotation system.

As shown in Fig. 5, we first imported the DICOM file of an image of whole-body SPECT bone and the diagnostic report into the LabelMe based annotation system. The three nuclear medicine doctors from our research group then manually labelled the areas on the image of the DICOM file (the RGB format is used currently) by using a shape tool such as rectangle and polygon in the available toolbar in the system. The labelled area was annotated with a self-defined symbol and the name of disease or body part. Manual annotations for all SPECT images are regarded as the ground truth in our experiments. An annotation file was finally formed together. The annotation file will be fed into the deep classification models.

Specifically, according to the diagnostic report, the annotation process of a thoracic image was performed by nuclear medicine doctors independently. If at least two doctors regarded an image as abnormal, i.e., at least one hot spot presents, it is labelled as an abnormal, and a normal otherwise. Note that in our dataset, an image that may contain multiple hot spots belongs to the same other than the different diseases.

### Deep learning-based classifiers

As an emerging and mainstream machine learning technique, deep learning has achieved great success in many areas such as machine vision and natural language processing in recent years. A number of deep architectures are available, such as the convolutional neural networks (CNNs)^21^, recurrent neural networks (RNNs)^22^, and deep belief networks (DBNs)^23^ as well as generative adversarial networks (GANs)^24^. In particular, CNNs automatically extract image features with different abstraction levels by using convolution operators. It is trained end-to-end in a supervised way.

CNNs are widely used in medical image analysis because of the use of sharing weights. Such sharing relies on the fact that similar structures reoccur in different locations within an image. In the computer-aided diagnosis (CAD), a CNNs-based classification system can automatically learn the features from images to form a function mapping inputs to outputs such as diseases present or absent. CNNs-based CAD systems are more popular than those systems relying on handcrafted features as in traditional machine learning.

In this work, we develop several deep classifiers based on the CNN models that are detailed below.

#### VGGNet based classifiers

To examine the effect of the convolutional network (ConvNet) depth on its accuracy in the large-scale image recognition task (e.g., ImageNet^25^), Simonyan and Zisserman^26^ developed deep several convolutional network architectures by pushing the depth to 11–19 weight layers, ranging from VGG-11, VGG-13 and VGG-16 to VGG-19. In the ConvNet configuration, the depth of the network was steadily increased through adding more convolutional layers that use 3 × 3 convolution filters in all layers while other parameters were fixed.

In this work, we use the standard VGG-16 and VGG-19 networks without modification to develop two classifiers, named *SPECS V16* (**SPE**CT **C**la**S**sifier with **V**GG-**16**) and *SPECS V19*, respectively. Meanwhile, in order to examine the effect of the ConvNet depth on accuracy in the thoracic image classification task, in this work we propose three different deep networks VGG-7, VGG-21 and VGG-24, corresponding to three classifiers, i.e., *SPECS V7*, *SPECS V21* and *SPECS V24*, by following the generic design of VGG-16 and VGG-19 networks. Table 3 outlines the configurations of the used VGGNets in this work.

**Table 3:** ConvNet configuration of VGGNets. The depth of the configurations increase from the left to the right, as more layers are added. The convolutional layer parameters are denoted as “conv <receptive file size> – <number of channels>.

SPECS V7	SPECS V16	SPECS V19	SPECS V21	SPECS V24
7 weight layers	16 weight layers	19 weight layers	21 weight layers	24 weight layers
**Input (256 × 256 Thorax SPECT image)**				
Conv3-64	Conv3-64	Conv3-64	Conv3-64	Conv3-64
Conv3-64	Conv3-64	Conv3-64	Conv3-64	Conv3-64
			Conv3-64	Conv3-64
**Maxpool**				
Conv3-128	Conv3-128	Conv3-128	Conv3-128	Conv3-128
Conv3-128	Conv3-128	Conv3-128	Conv3-128	Conv3-128
			Conv3-128	Conv3-128
	**Maxpool**			
	Conv3-256	Conv3-256	Conv3-256	Conv3-256
	Conv3-256	Conv3-256	Conv3-256	Conv3-256
	Conv3-256	Conv3-256	Conv3-256	Conv3-256
		Conv3-256	Conv3-256	Conv3-256
				Conv3-256
	**Maxpool**			
	Conv3-512	Conv3-512	Conv3-512	Conv3-512
	Conv3-512	Conv3-512	Conv3-512	Conv3-512
	Conv3-512	Conv3-512	Conv3-512	Conv3-512
		Conv3-512	Conv3-512	Conv3-512
				Conv3-512
	**Maxpool**			
	Conv3-512	Conv3-512	Conv3-512	Conv3-512
	Conv3-512	Conv3-512	Conv3-512	Conv3-512
	Conv3-512	Conv3-512	Conv3-512	Conv3-512
		Conv3-512	Conv3-512	Conv3-512
				Conv3-512
MaxPool				
FC-4096				
FC-4096				
FC-2				
Soft-max				

In the VGG network architectures, the *input* to the ConvNets is a fixed-size 256 × 256 thoracic image that passes through a stack of convolutional (conv.) layers with 3 × 3 filters used. The convolution *stride* is fixed to 1 pixel while the *padding* is 1 pixel for 3 × 3 conv. layers. Spatial pooling uses five *max-pooling* layers over a 2 × 2 pixel window with a stride of 2. Some conv. Layers are then followed. All hidden layers use the ReLU rectification non-linearity (except for the SPECS V7). The ReLU function is defined in Eq. (2).

2 $$ {\text{ReLU}}\;(x) = \left\{ {\begin{array}{*{20}l} {x,} & {if\;x \ge 0} \\ {0,} & {if\;x < 0} \\ \end{array} } \right. $$

A stack of conv. layers is followed by the three Fully-Connected (FC) layers: the first two have 4096 channels each, the third performs 2-way metastasis classification and thus contains 2 channels (one for each class). The final layer is the soft-max layer. The configuration of the fully connected layers is the same in all networks.

The L2 regularization is introduced in VGG-7 to reduce overfitting, which is defined as follows.

3 $$ L = e + \lambda \sum\limits_{j} {w_{j}^{2} } $$

where *e* is the training error if regularization is not used in the training process, and *λ* is the regularization parameter.

#### ResNet based classifier

**Figure 6:**
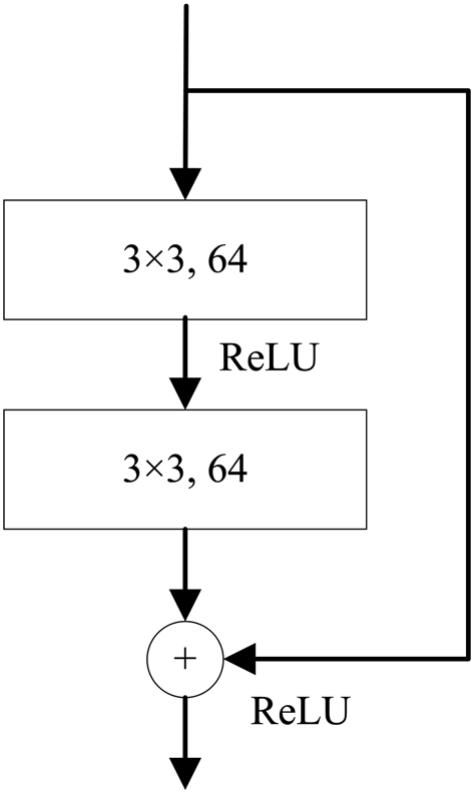
The structure of residual module in ResNet-34 network.

The ResNet^27^ was proposed to deal with the poor generalization problem in a deeper network by introducing residual modules into the network. Specifically, ResNet-34 is chosen as the basis to develop our classifier, in which each residual module consists of two conv. layers and one residual connection (see Fig. 6). A total of sixteen residua modules (3, 4, 6, 3) are contained in our ResNet-34 based classifier.

In the network, a stack of conv. layers with 3 × 3 filters are used, followed by an FC layer. Spatial pooling is carried out by one max-pooling layer over a 3 × 3 pixel window with stride 2 and an average pooling over a 2 × 2 pixel window with stride 1. The classifier built based on RestNet-34 is named as *SPECS R34* in this paper.

#### DenseNet based classifier

The Dense Convolutional Network (DenseNet) proposed by Huang et al.^28^ differs from the ResNet by concatenating rather than adding features to reduce the computational burden. DenseNet has the potential to alleviate the vanishing gradient problem, strengthen feature propagation and encourage feature reuse.

Table 4 provides the ConvNet configuration of DenseNet, in which the input to the ConvNet is a thoracic image with the fixed-size of 256 × 256 that will be passed through a stack of conv. layers and four dense blocks. Spatial pooling is carried out by a max-pooling layer, three average pooling layers and a global average pooling (GAP) layer. All hidden layers are equipped with the ReLU rectification non-linearity. The FC layer has 2 channels each (one for each class, i.e., normal and metastasis). The final layer is the softmax function.

**Table 4:** ConvNet configuration of DenseNet.

SPECS D121	
121 weight layers	
input (256 × 256 thoracic image)	
7 × 7 conv.	
3 × 3 Max pooling	
Dense Block (1)	$$\left[ {\begin{array}{*{20}l} {1 \times 1\;{\text{ conv}}.} \\ {3 \times 3 \, \;{\text{conv}}.} \\ \end{array} } \right] \times 6$$
1 × 1 conv.	
2 × 2 Average pooling	
Dense Block (2)	$$\left[ {\begin{array}{*{20}l} {1 \times 1\;{\text{ conv}}.} \\ {3 \times 3 \, \;{\text{conv}}.} \\ \end{array} } \right] \times 12$$
1 × 1 conv.	
2 × 2 Average pooling	
Dense Block (3)	$$\left[ {\begin{array}{*{20}l} {1 \times 1 \, \;{\text{conv}}{.}} \\ {3 \times 3\;{\text{ conv}}.} \\ \end{array} } \right] \times 24$$
1 × 1 conv.	
2 × 2 Average pooling	
Dense Block (4)	$$\left[ {\begin{array}{*{20}l} {1 \times 1\;{\text{ conv}}{.}} \\ {3 \times 3\;{\text{ conv}}{.}} \\ \end{array} } \right] \times 16$$
7 × 7 Global average pooling	
FC-2	
Soft-max	

**Figure 7:**
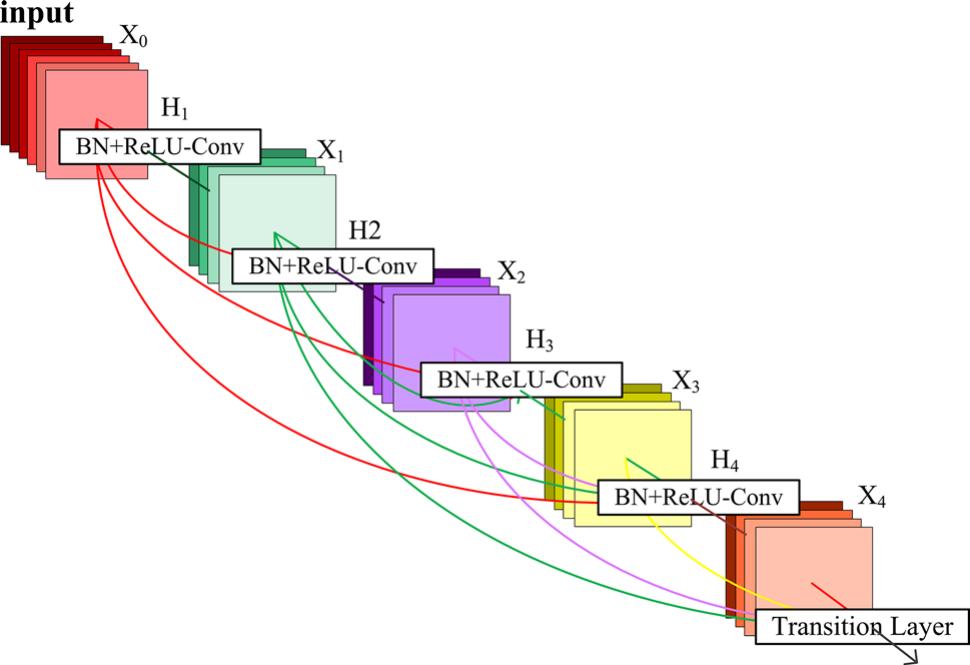
The structure of dense block in DenseNet-121 network.

Each layer within each dense block denoted by X_*i*_ (see Fig. 7) is connected to each of other layers in a feed-forward way. The feature maps of all preceding layers in each layer, together with its own feature-maps, are used as inputs into all subsequent layers. H_*i*_ is a combination of non-linear transformation including a batch normalization (BN), a ReLU and a pooling. A transition layer consisting of a 1 × 1 conv. layer and a 2 × 2 average pooling layer is used to connect two adjacent dense blocks. The classifier built based on DenseNet-121 is named as *SPECS D121* in this paper.

In summary, we develop seven different deep classifiers, i.e., SPECS V7, SPECS V16, SPECS V19, SPECS V21, SPECS V24, SPECS R34 and SPECS D121. For each of the defined classifiers, the parameters in the whole network are randomly initialized using a normal Gaussian distribution to increase the network robustness. All these classifiers will be experimentally evaluated by using real-world thoracic images in the following section.

## Results

In this section, we report the empirical evaluation of the proposed deep classifiers against real-world thoracic images from three different datasets, i.e., *D*_1_–*D*_3_.

### Experimental setup

The evaluation metrics are accuracy, precision, recall, *F*-1 score, specificity and AUC (Area Under ROC Curve). A given image is classified into one of the following four categories:

- True Positive (*TP*), correctly predicts an abnormal image as positive;
- False Positive (*FP*), incorrectly predicts a normal image as positive;
- False Negative (*FN*), incorrectly predicts an abnormal image as normal; and
- True Negative (*TN*), correctly predicts a normal image as normal.

Accordingly, we define accuracy (*Acc*), precision (*Prec*), recall (*Rec*), specificity (*Spe*), and *F*-1 score in Eqs. (4)–(8).

4 $$ Acc = \frac{TP + TN}{{TP + TN + FP + FN}} $$

5 $$ Prec = \frac{TP}{{TP + FP}} $$

6 $$ Rec = \frac{TP}{{TP + FN}} $$

7 $$ Spe = \frac{TN}{{TN + FP}} $$

8 $$ F - 1 = 2 \times \frac{Prec \times Rec}{{Prec + Rec}} $$

Desirably, a classifier should have both a high true positive rate (*TPR* = *Rec*) and a low false-positive rate (*FPR*) at the same time. The ROC curve plots the true positive rate (*y*-axis) against the false positive rate (*x*-axis). The AUC value is defined as the area under the ROC curve. As a statistical explanation, an AUC score is equal to the probability that a randomly chosen positive image is ranked higher than a randomly chosen negative image. Thus, the closer to 1 the AUC value is, the higher performance the classifier performs. The false-positive rate (*FPR*) is defined in Eq. (9).

9 $$ FPR = \frac{FP}{{FP + TN}} = \frac{TN}{{TN + FP}} $$

Each dataset (i.e., *D*_1_, *D*_2_, and *D*_3_) is randomly divided into two parts, i.e., training subset and test subset. The ratio of the training subset and the test subset is 7:3. The samples in the training subsets are used to train the classifiers while the samples in the test subsets are used to test the classifiers. A trained classifier was run 10 times on the test subset in order to reduce the effects of randomness. For each of the defined metrics above, the final output of a classifier is the average of these 10 results. The experimental results reported in the forthcoming section are the averaged ones unless otherwise specified.

The parameters settings are reported in Table 5.

**Table 5:** Parameters setting of the developed deep classifiers.

Parameter	Value
Learning rate	0.001
Optimizer	Batch gradient descent (BGD)
Batch size	32
Epoch	500

The experiments are run in Tensorflow 2.0 on an Intel Core i7-9700 PC with 32 GB RAM running Windows 10.

### Experimental results

In the experiments, we examine the performance of deep classifiers on three different datasets, i.e., the original dataset *D*_1_, the augmented dataset with normalization *D*_2_, and the augmented dataset without normalization *D*_3_.

**Figure 8:**
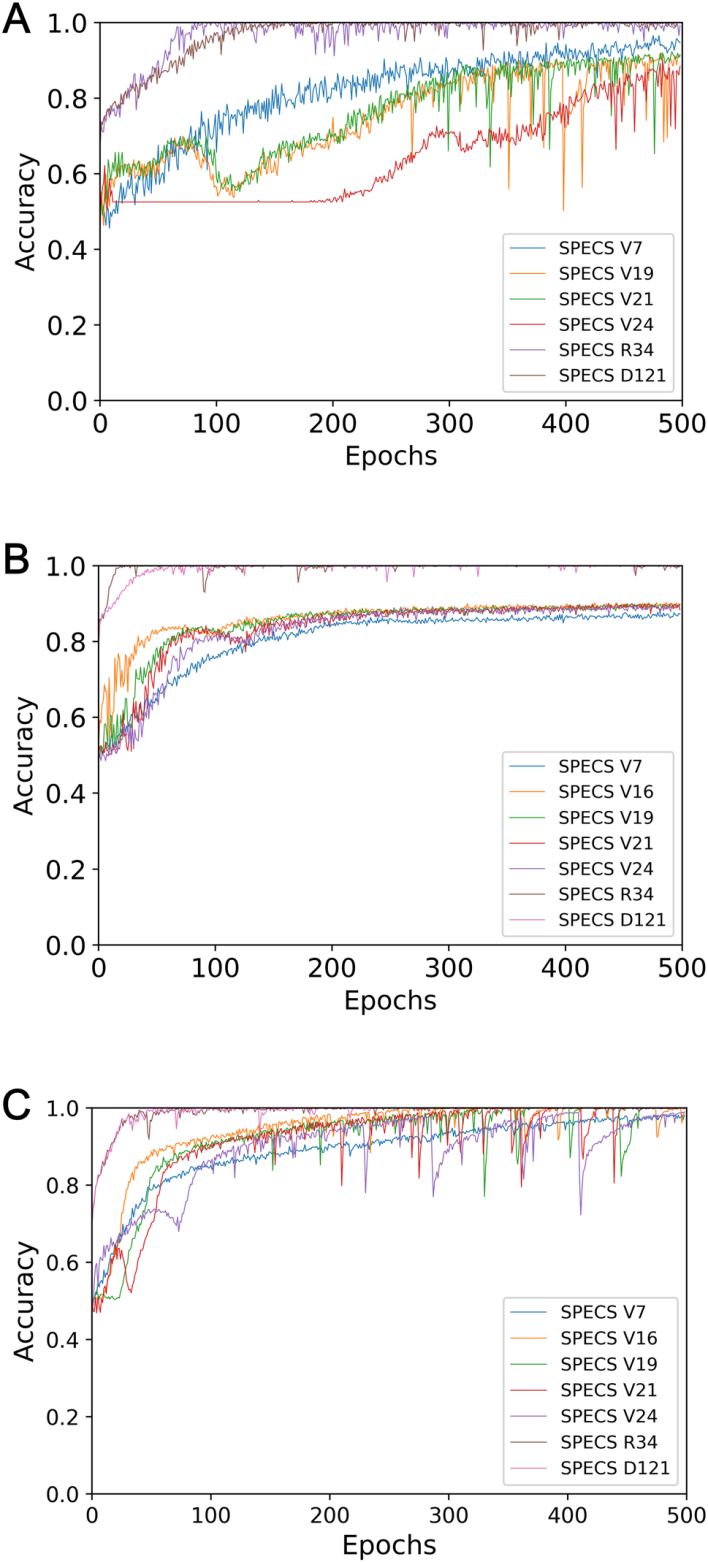
The accuracy curves obtained by training classifiers on training subsets in different datasets. (**a**) Dataset *D*_1_; (**b**) Dataset *D*_2_; and (**c**) Dataset *D*_3_.

On the accuracy metric, Fig. 8 depicts the training processes of our seven different classifiers over the training subsets in *D*_1_, *D*_2_, and *D*_3_, respectively.

From the accuracy curves as depicted in Fig. 8 we can see that: (1) all classifiers obtain better performance on the augmented datasets than on the original one; (2) the proper depth of the network architecture is recommended for high performance; and (3) data normalization has no contribution on the classification performance for deep classifiers on our augmented dataset. This can be further proved by the quantitative results of evaluation metrics on test samples in Tables 6, 7, 8.

**Table 6:** Experimental results on evaluation metrics for test samples in dataset *D*_1_.

Classifier	*Acc*	*Prec*	*Rec*	*Spe*	*F*-1 *score*
SPECS V7	0.7928	0.6875	0.9361	0.6875	0.7928
SPECS V16	0.8288	0.9667	0.6170	0.9843	0.7532
SPECS V19	0.8829	0.8542	0.8723	0.8906	0.8632
SPECS V21	0.9190	0.9750	0.8298	0.9844	0.8966
SPECS V24	0.7387	0.6731	0.7447	0.7343	0.7070
SPECS R34	0.8198	0.8462	0.7021	0.9063	0.7674
SPECS D121	0.8018	0.8209	0.6809	0.8906	0.7442

**Table 7:** Experimental results on evaluation metrics for test samples in dataset *D*_2_.

Classifier	*Acc*	*Prec*	*Rec*	*Spe*	*F*-1 *score*
SPECS V7	0.8508	0.9020	0.7817	0.9176	0.8376
SPECS V16	0.8605	0.8799	0.8300	0.8901	0.8542
SPECS V19	0.8410	0.8329	0.8470	0.8352	0.8399
SPECS V21	0.8800	0.9495	0.7989	0.9588	0.8677
SPECS V24	0.8397	0.9075	0.7507	0.9258	0.8217
SPECS R34	0.8787	0.9030	0.8442	0.9121	0.8726
SPECS D121	0.9317	0.9634	0.8952	0.9670	0.9280

**Table 8:** Experimental results on evaluation metrics for test samples in dataset *D*_3_.

Classifier	*Acc*	*Prec*	*Rec*	*Spe*	*F*-1 *score*
SPECS V7	0.8059	0.9240	0.7932	0.9366	0.8536
SPECS V16	0.9679	0.9824	0.9518	0.9835	0.9669
SPECS V19	0.9791	0.9829	0.9745	0.9835	0.9787
SPECS V21	0.9807	0.9775	0.9830	0.9780	0.9802
SPECS V24	0.9553	0.9848	0.2350	0.9862	0.5320
SPECS R34	0.8842	0.9856	0.7762	0.9890	0.8685
SPECS D121	0.7824	0.9900	0.5637	0.9945	0.7184

**Figure 9:**
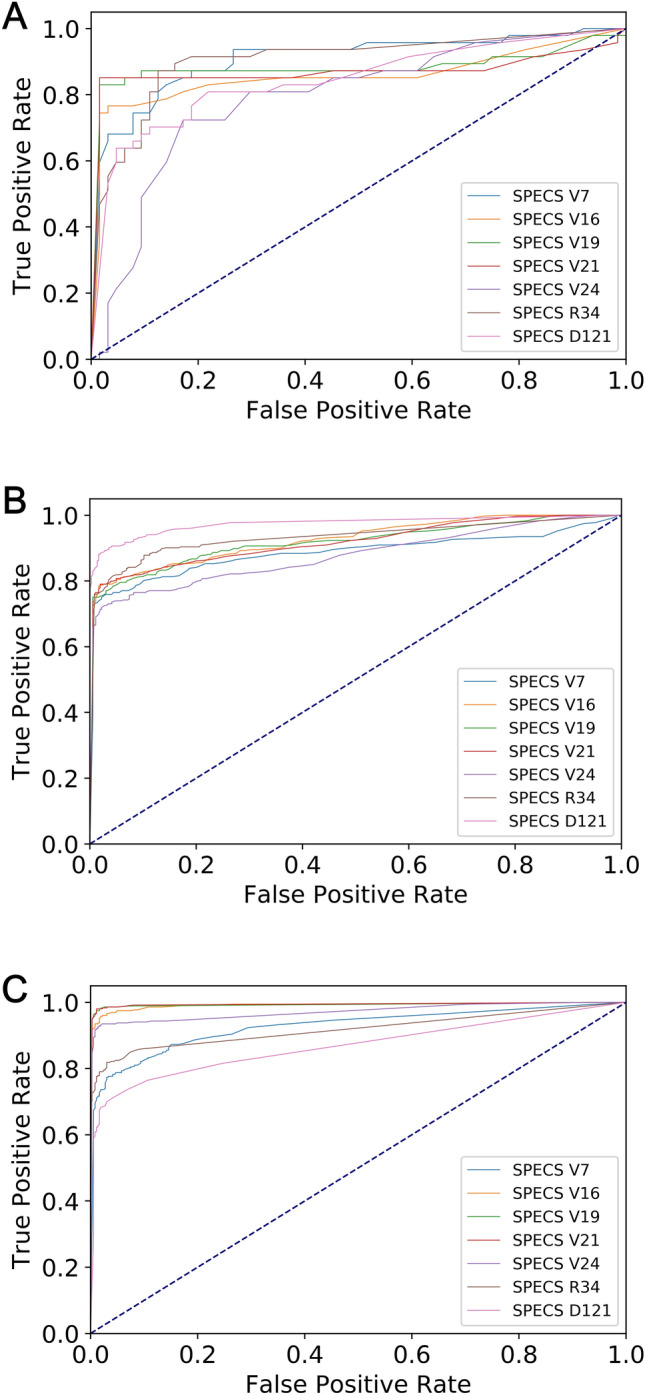
The ROC curves of the classifiers on test samples in different datasets. (**a**) Dataset *D*_1_; (**b**) Dataset *D*_2_; and (**c**) Dataset *D*_3_.

The self-defined 21-layer classifier SPECS V21 based on VGG is suitable for identifying bone metastasis with SPECT imaging, obtaining a value over 0.98 for all metrics (see Table 8). The ROC curves depicted in Fig. 9 illustrate the true positive rate and the false positive rate simultaneously. The corresponding AUC values are provided in Table 9. The self-defined classifier SPECS V21 achieves the highest AUC value of 0.993.

**Table 9:** AUC values obtained by classifiers on test samples in different datasets.

Dataset	SPECS V7	SPECS V16	SPECS V19	SPECS V21	SPECS V24	SPECS R34	SPECS D121
*D*_1_	0.9069	0.8562	0.8820	0.8688	0.7931	0.9034	0.8443
*D*_2_	0.8860	0.9255	0.9176	0.9181	0.8768	0.9324	0.9745
*D*_3_	0.9267	0.9909	0.9920	0.9933	0.9713	0.9186	0.8677

**Figure 10:**
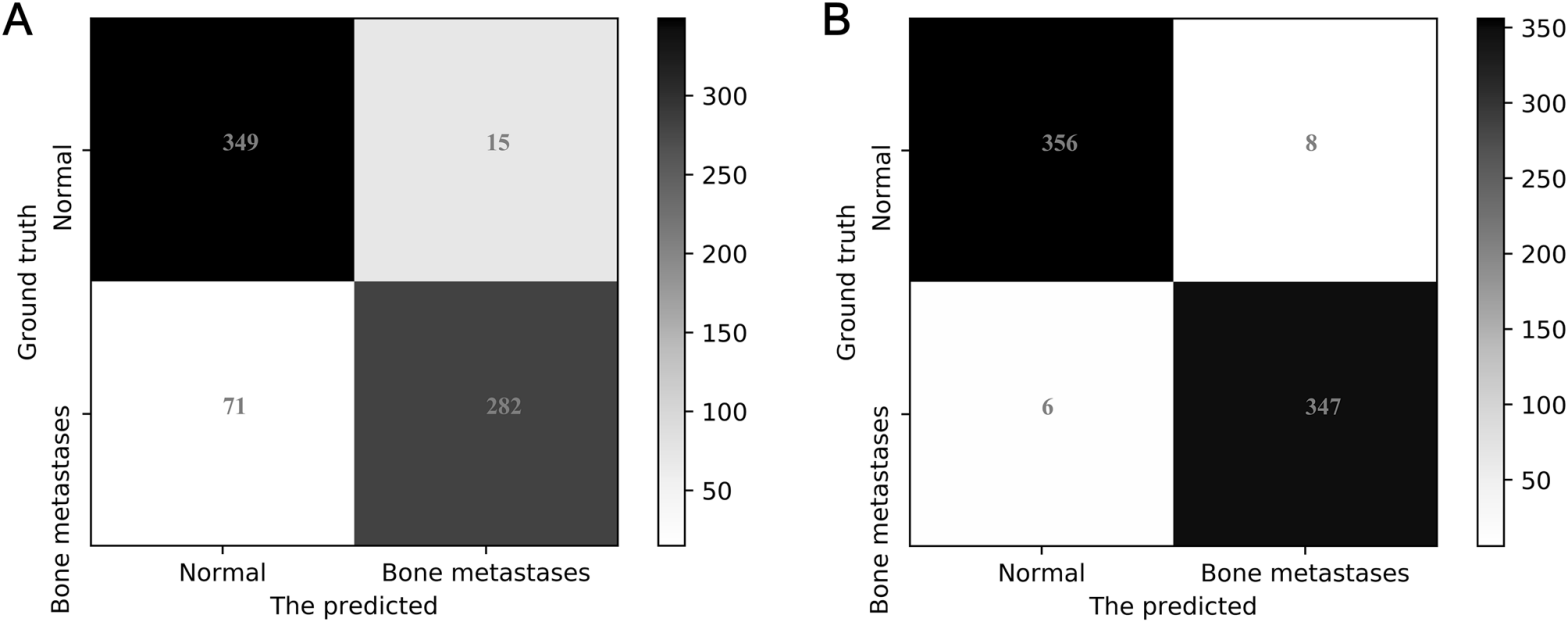
The confusion matrixes obtained by SPECS V21 on: (**a**) Dataset *D*_2_; and (**b**) Dataset *D*_3_.

We further examine the performance of classifiers on differentiating various subcategories (i.e., the normal and metastasized) of thoracic images by providing the confusion matrixes of SPECS V21 on datasets *D*_2_ and *D*_3_, which are depicted in Fig. 10. From which we can see that a significant proportion of samples of metastasized images were misclassified as normal by SPECS V21 on the augmented dataset with normalization. On contrast, only 6 metastasized images and 8 normal images were misclassified by the classifier SPECS V21 on the augmented dataset without normalization. We can thus conclude that the size of the dataset is critical for the performance of deep learning-based SPECT image classification.

**Figure 11:**
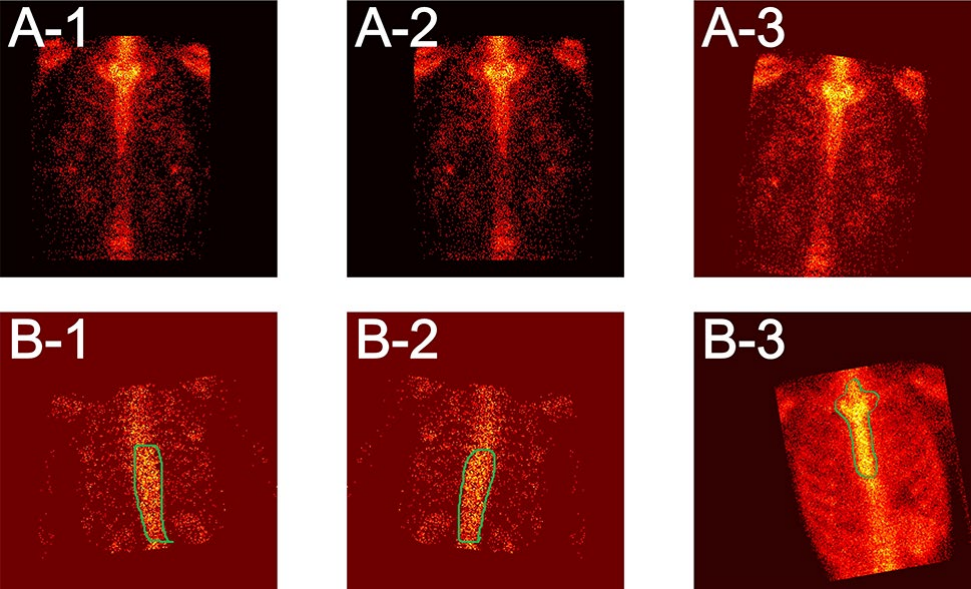
A demonstration of misclassified thoracic SPECT bone images. (**a**) Misclassified normal images as the metastasized; and (**b**) misclassified metastasized images as the normal.

Now, we provide a brief discussion on the misclassified images by SPECS V21 provided in Fig. 11 as follows.

- Random rotation significantly contributes to augmentation of the dataset while introducing errors in the receptive field (a small area of an image) between images for deep networks. Therefore, the rotation operation is mainly in charge of both the misclassified normal and metastasized images.
- Difference in radiation dosage from person to person requests more personalized features to be extracted from a big dataset of SPECT bone images. For SPECT imaging, the absorption of radionuclide is inversely proportional to patient age, making metastasis images to be misclassified as normal. Therefore, some post-processing operations should be conducted after automatic classification, by taking the structural symmetry of human bones into consideration.

In conclusion, the developed deep classifiers have successfully identified the metastasized images, obtaining high sensitivity and specificity simultaneously. However, the size of the dataset is crucial for training a reliable and accurate deep classifier. Data augmentation contributes to extending the size of datasets but further improvements would be gained through introducing, for example, the generative adversarial networks to generate those new but different samples.

## Conclusions

Targeting at the automated diagnosis of bone metastasis in SPECT nuclear medicine domain, in this work, we have developed several deep classifiers based on the famous CNN models to separate the thoracic SPECT bone images into the categories with the automatically extracted features by deep learning models. Specifically, the original SPECT images was preprocessed by using standard image mirroring, translation, and rotation operations, enabling to generate an augmented dataset. Famous CNN models were introduced to develop deep classifiers, which could classify an image by analyzing from the low level to high level features from this image. A set of real-world SPECT bone images were employed to evaluate the trained classifiers. The experimental results have demonstrated that our deep classifiers perform well on classifying thoracic SPECT bone images, with huge potentials to automatically diagnose bone metastasis in SPECT imaging.

Our future work is in the following directions. First, a larger number of images of real-world SPECT bone scans will be collected to further evaluate the proposed deep classifiers. Second, we attempt to develop multi-class, multi-disease classifiers to identify lesions of various diseases with SPECT bone images. Finally, we will construct self-defined deep networks, specifically targeting at the automated classification of SPECT bone images for contributing to the current research of medical image analysis.

## Data Availability

Due to the ethical and legal restrictions on the potential health information of patients, the data are not available openly. Dataset can only be accessed upon request by emailing the co-author Haijun Wang (Email: 1718315929@qq.com) who is on behalf of the Ethics Committee of Gansu Provincial Hospital.
